# Controlling Molecule Aggregation and Electronic Spatial Coherence in the H-Aggregate and J-Aggregate Regime at Room Temperature

**DOI:** 10.3390/polym12040786

**Published:** 2020-04-02

**Authors:** Fei Dou, Jiawei Li, Huijie Men, Xinping Zhang

**Affiliations:** Institute of Information Photonics Technology and College of Applied Sciences, Beijing University of Technology, Beijing 100124, China; doufei@bjut.edu.cn (F.D.); S201706087@emails.bjut.edu.cn (J.L.); 17854254186@163.com (H.M.)

**Keywords:** H-aggregate, J-aggregate, electronic spatial coherence, spectroscopy

## Abstract

Controlling molecule aggregation in polymer films is one of the key factors in understanding the links between properties and structures in organic semiconductors. Here, we used poly(3-hexylthiophene-2,5-diyl) (P3HT) as the model system. By doping the insulating polar additive poly (ethylene oxide) (PEO) into P3HT film and controlling the processing methods, we achieved the side-to-side H-aggregate and head-to-tail J-aggregate of P3HT molecules with different extents at room temperature. We have demonstrated that the solvent solidification rate plays an important role in the controlling of molecule aggregation, which finally influenced the solid-state phase separation in the film. Furthermore, based on a series of spectroscopy investigations, we quantified the electronic spatial coherence in different aggregations combined with the modified Franck–Condon model. Subsequently, we established the relationship between the processing method, the molecule aggregation, and the electronic spatial coherence.

## 1. Introduction

The device performance of polymer solar cells is strongly dependent on blend structures. Understanding the links between blend properties and film structures is fundamentally important in organic semiconductors and has attracted lots of attention to research, especially at the molecular level. Aggregation is fundamental to the elastic polymer, and represents the short-range ordering of molecules along the polymer chain (J-aggregate) and across the polymer chain (H-aggregate) on the molecular scale. Recently, many pieces of research have connected molecular aggregation to film properties and reinforced the influence of molecule aggregation on the material’s applicability in solar cells [1,2].

Prof. Yan and Prof. Ade’s collaborative work found that aggregation and morphology control enables high-efficiency thick-film polymer solar cells, where over 10% high-efficiency has been yielding for three different donor polymers and 10 polymers: fullerenes combination [3]. It has also been demonstrated that the molecule aggregation of donor polymers can be tuned by side-chain engineering, and enhanced polymer solar cell efficiency can be achieved [1,4,5,6]. Prof. Ade and Prof. Hou’s collaborative work demonstrated that donor polymer aggregation can be optimized to improve ordering structure and pi–pi interactions, leading to a 14.2% high-efficiency polymer solar cell [7]. However, a fundamental understanding of the interlink between molecule packing or aggregation with their electronic properties is still lacking in the semiconductor field. In addition, the material aggregation has not been systematically controlled in both H- and J-aggregation regimes.

It has been demonstrated that molecule aggregation of polymer films can be modified under specific experimental conditions, such as changing the molecular weight [8], doping the insulating material [9], adding the alkyl-chain [10], irradiation by ultraviolet light [11], changing the side-chain structure [12], changing the deposition temperature [13], and changing the transition temperature [14]. Specifically, C. Hellmann et al. found that the poly (3-hexylthiophene-2, 5-diyl) (P3HT) backbone can be more planar (J-aggregate behavior) by doping polyethylene oxide (PEO) into P3HT film, and there is a transition from H-aggregate to J-aggregate of P3HT molecules by casting the film below 18 °C [9]. It has also been suggested that spin-coating film below the critical temperature (−2 °C) can enhance the formation of aggregates with strong intrachain coupling in P3HT film [13].

In this work, we firstly systematically controlled the aggregation of P3HT molecules in both H- and J-aggregate regimes through doping insulating polar additive PEO into the film at room temperature. Then, we combined several spectroscopy techniques and the modified Franck–Condon model to characterize the molecular ordering, electronic spatial coherence, and exciton dynamics in different aggregation. In the end, we revealed the relationship between processing methods, molecule aggregation, and electronic spatial coherence in H- and J-aggregate regimes of P3HT film.

## 2. Experimental Methods

For thin-film fabrication, P3HT (*M*_n_ = 38 kg/mol) and PEO (*M*_n_ = 900 kg/mol) were codissolved (60 wt % PEO) in chloroform with the concentration of 16 mg/mL at 55 °C. When the solution was totally dissolved, we spun coated (or wire-bar-coated) the codissolved hot solution on quartz substrates at room temperature (25 °C) with different spin-coating rates for 20 s (or with different bar size at the speed of 200 mm/min). In order to enhance the polarity of PEO, we immersed the spun coated or wire-bar-coated film in the deionized (DI) water for 60 s when the film was totally dried.

To understand the molecule aggregation and exciton coherence of the fabricated films, we operated a series of spectroscopy techniques. It included UV-Vis absorption spectroscopy (Agilent Technologies G1103A, Agilent Technologies, Santa Clara, CA, USA), optical microscopic spectroscopy (Olympus BX51, Olympus Corporation, Tokyo, Japan), low-temperature photoluminescence (PL) spectroscopy (*T* = 10 K, Advanced research systems, Macungie, PA, USA), and time-resolved PL (Tr-PL) spectroscopy (FL920, Edinburgh Instrument Ltd., Scotland, UK). In the 10 K PL measurements, the sample was mounted in a closed-cycle cryostat with the exchange gas at 10 K.

## 3. Results and Discussion

### 3.1. Controlling of the Molecule Intrachain Ordering in the H-aggregate Regime

**Figure 1:**
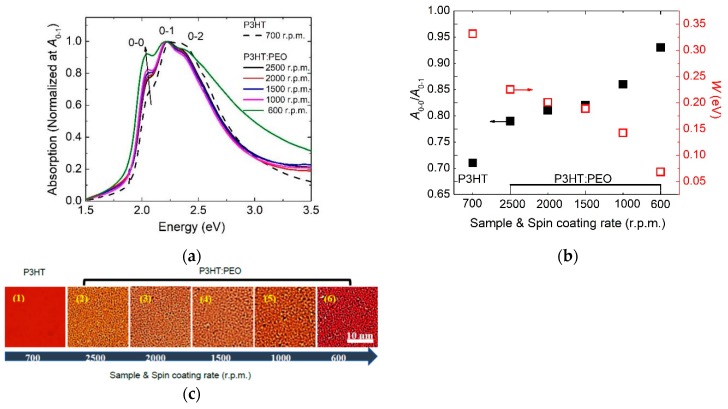
(**a**) UV-Vis absorption spectra of poly (3-hexylthiophene-2,5-diyl) (P3HT) and P3HT: poly (ethylene oxide) (PEO) film with different spin-coating rates. 0-0, 0-1, and 0-2 vibronic features reflect transitions from the ground state to the first excited state. All spectra are normalized at the 0-1 peak. (**b**) The vibronic transition ratio *A*_0-0_/*A*_0-1_ and *W* are deduced from (**a**) and plotted as a function of the spin-coating rate. (**c**) Optical microscopic images of P3HT and P3HT: PEO film with different spin-coating rates.

We firstly focus our efforts on the dependence of the spin-coating rate on the molecule aggregation of P3HT film. The different aggregations are analyzed by UV-Vis absorption spectroscopy, shown in Figure 1a. We normalize all spectra of the *A*_0-1_ absorption peak, in order to compare the relative intensity of the aggregation-involved *A*_0-0_ electronic transition with *A*_0-1_. The dependence of *A*_0-0_/*A*_0-1_ on samples and spin-coating rates is plotted in Figure 1b.

Interestingly, it is clear that the *A*_0-0_/*A*_0-1_ ratio of P3HT: PEO film is enhanced compared with P3HT film, indicating that intrachain aggregation of P3HT molecules can be enhanced by using PEO as the additive. The increased *A*_0-0_/*A*_0-1_ ratio suggests that P3HT molecules adopt more ordered intrachain packing in P3HT: PEO film than the P3HT film. This finding agrees with the reported work that the addition of PEO can increase the intrachain molecule aggregation in P3HT film. In the reported work, the role of PEO on the aggregation of P3HT molecules relies on the initial liquid–liquid phase separation, and the liquid–liquid phase separation has been modified by cooling down the fabrication temperatures [12]. Furthermore, decreasing the spin-coating rate of P3HT: PEO films (from 2500 r.p.m. down to 600 r.p.m.), the *A*_0-0_/*A*_0-1_ ratio increased sharply from 0.79 up to 0.93, indicating that when reducing the spin-coating rate, the molecule intrachain ordering can be effectively enhanced in P3HT: PEO films.

Quantitatively, according to the weakly interchain-coupled modified Franck–Condon model [15], we calculate the free-exciton bandwidth *W* in these films based on the achieved *A*_0-0_/*A*_0-1_ ratio,

(1) $$\frac{A_{0-0}}{A_{0-1}}\approx {\left(\frac{1-0.24W/\hbar \omega_{0}}{1+0.073W/\hbar \omega_{0}}\right)}^{2}$$

where *A*_0-0_/*A*_0-1_ is related oscillator strength of *A*_0-0_ and *A*_0-1_ vibronic progression, and $\omega_{0}$ is the frequency of the vibrational mode coupled to the electronic transition, which has been found at 0.18 eV in P3HT film [15]. Thus, we can use Equation (1) to calculate interchain free-exciton bandwidth *W*, and the values are shown in Figure 1b.

Clearly, when the *A*_0-0_/*A*_0-1_ ratio increasing (from 0.71 up to 0.93), *W* decreased (from 0.33 eV down to 0.07 eV) as the function of spin-coating rates. The decreased interchain free-exciton bandwidth indicates the decrease of the interchain coupling or the increase of the intrachain ordering in P3HT: PEO spin-coated film. It is suggested that the intrachain ordering can be increased in the weakly coupled H-aggregate regime of P3HT molecules by reducing the spin-coating rate during the film fabrication.

In order to understand why there is a strong dependence of molecule aggregation on spin-coating rates, we use the microscopic optical spectroscopy to characterize the surface morphology of these films, which are captured by the transmission mode. Images are shown in Figure 1c, where the P3HT is present in orange or red depending on the film thickness, and the PEO is present in white due to the weak absorption. We have found that the surface of the P3HT film is smooth and homogeneous, namely, without obvious cluster or phase separation. However, all P3HT: PEO films show very rough surfaces, and the phase separation increased with the decrease of the spin-coating rates, indicating that the ordered intrachain are generated more on phase-separated microstructures. Thus, we speculate that phase separation is beneficial for the formation of the intrachain ordering of P3HT molecules, which can be increased by the reduction of the solvent solidification rate.

### 3.2. Controlling of the Molecule Intrachain Ordering in the J-Aggregate Regime

**Figure 2:**
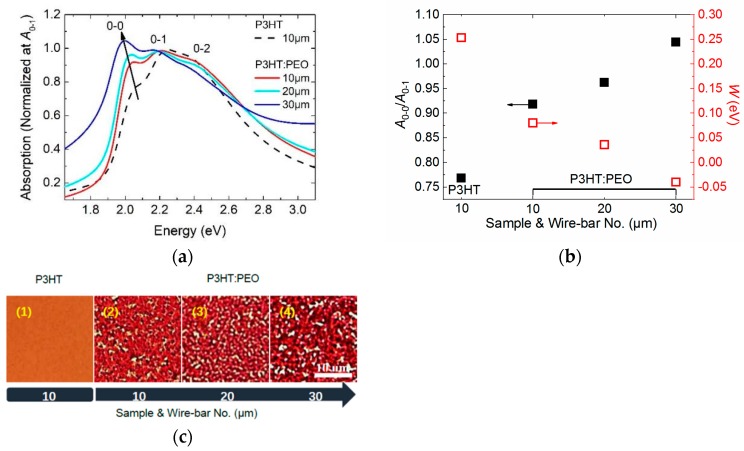
(**a**) Absorption spectra of poly (3-hexylthiophene-2,5-diyl) (P3HT) and P3HT: poly (ethylene oxide) (PEO) film prepared by wire-bar coating with various bars. The *A*_0-0_, *A*_0-1_, and *A*_0-2_ vibronic features reflect transitions from the ground state to the first excited state. Absorption spectra are normalized at *A*_0-1_. (**b**) Vibronic transition ratios *A*_0-0_/*A*_0-1_ and the interchain exciton bandwidth *W* are plotted as a function of bar sizes. *A*_0-0_/*A*_0-1_ are deduced from the absorption spectra, and interchain exciton bandwidth *W* is calculated by Equation (1). (**c**) Optical microscopy images of P3HT and P3HT: PEO film coated with different bars.

To demonstrate our speculation, we change the film processing method from spin-coating to wire-bar coating in order to reduce the solvent solidification rate. Figure 2a shows the UV-Vis absorption spectra of film coated with different bars. Spectra are normalized at the *A*_0-1_ absorption peak in order to compare the relative intensity of the aggregation involved *A*_0-0_ electronic transition with *A*_0-1_. The dependence of *A*_0-0_/*A*_0-1_ on samples and bar sizes is plotted in Figure 2b (left axis).

We found that the *A*_0-0_/*A*_0-1_ ratio of P3HT: PEO film significantly increased compared with that of P3HT film, which increased from 0.77 to 0.91, whilst, as the bar size increasing from 10 μm to 30 μm, the *A*_0-0_/*A*_0-1_ ratio further increased from 0.91 to 1.04, along with a clear redshift of the *A*_0-0_ absorption. This increased oscillator strength and redshifted absorption of *A*_0-0_ indicates that the intrachain aggregation can be further enhanced in the wire-bar-coated film. Especially for 30 μm-bar coated film, the *A*_0-0_/*A*_0-1_ ratio is larger than 1, implying that the intrachain aggregation dominates the molecule packing in the film and the molecules adopt the head-to-tail J-aggregate packing. Thus, reducing the solidification rate of the film fabrication, we can both increase the molecule intrachain ordering in the side-to-side H-aggregate regime (the spin-coated film) and in the head-to-tail J-aggregate regime (the wire-bar-coated film).

Quantitatively, we calculated the free-exciton bandwidth *W* of different films using Equation (1) and plotted the *W* as a function of bar sizes shown in Figure 2b. Interestingly, we found that *W* decreased from 0.25 eV to −0.04 eV as the size of the bar increased. The value of *W* changed from positive to negative, indicating a transformation from the H-aggregate molecule packing (*W* > 0) to the J-aggregate molecule packing (*W* < 0). H- to J-aggregation transition of P3HT: PEO film has been observed under a very low-temperature circumstance (−2 °C) by Markus Reichenberger. [9]

According to the modified Franck–Condon model, we can establish a relationship between molecule aggregation and free-exciton bandwidth *W*. Specifically, in the H- or J-aggregate regime, the electronic coherence across the chain or along the chain is determined by the nearest-neighbor coupling *J*_0_, which is represented as the interband mixing and is related to the free-exciton bandwidth *W* (*W* = 4*J*_0_) [15]. In the H-aggregate regime, the nearest-neighbor chromophores are guided in a side-by-side manner. The molecular nucleus spacing becomes larger after electrons transition from the ground state to the first excitation state, leading to a strong allowed *A*_0-1_ transition and relatively forbidden *A*_0-0_ transition [16] (*A*_0-0_/*A*_0-1_ < 1), where *J*_0_ > 0.

However, in the case of the J-aggregate, the nearest-neighbor chromophores are directed in a head-to-tail manner. The spacing of the molecular nucleus remained nearly unchanged after electron transition from the ground state to the first excitation state, leading to a strongly allowed *A*_0-0_ transition and relatively weak *A*_0-1_ transition (*A*_0-0_/*A*_0-1_ > 1), where *J*_0_ < 0. Thus, the positive electronic coupling (*W* > 0) in spin-coated film denotes P3HT molecules adopting H-aggregate packing and the negative electronic coupling (*W* < 0) in the wire-bar-coated film indicates P3HT molecules adopting J-aggregate packing.

Figure 2c shows the optical microscopic images of P3HT and P3HT: PEO film, which are coated with different bars, and the images are taken in transmission mode. Similarly, in the spin-coated film, all P3HT: PEO films present phase-separated surfaces, with clear red P3HT domains and white PEO domains. However, in contrast to the spin-coated film, wire-bar coated film represents a larger domain size, implying that the wire bar-coated films are more phase-separated. In addition, when we decrease the solvent solidification rate (increasing bar sizes), the film displays increased domain sizes, implying enhanced phase separations.

Therefore, the strong dependence of molecule aggregation on film solidification rate has been demonstrated for both spin-coated film and wire-bar-coated film. Decreasing the film solidification rate from liquid to solid state can enhance the solid-state phase separation of the film. As a result, the molecule intrachain ordering has been manipulated.

### 3.3. Exciton Generations in H- and J-Aggregate Regimes

**Figure 3:**
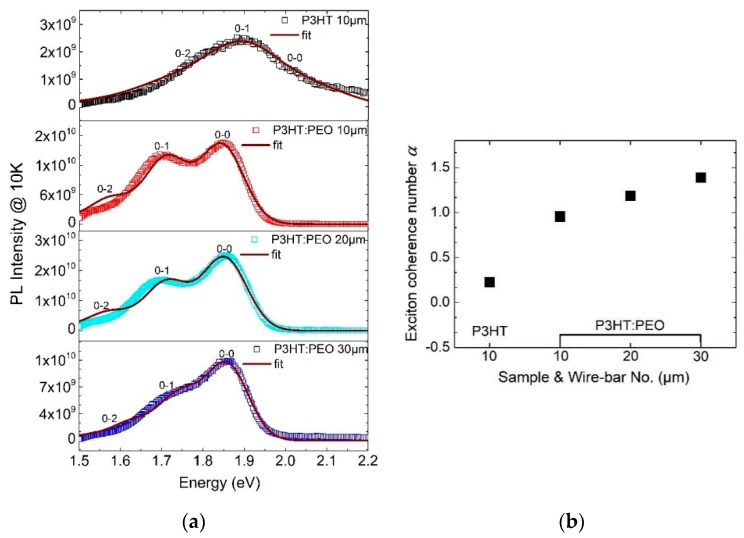
(**a**) PL spectra of poly (3-hexylthiophene-2,5-diyl) (P3HT) and P3HT: poly (ethylene oxide) (PEO) wire-bar-coated film with the excitation at 470 nm. *I*_0-0_, *I*_0-1_, *I*_0-2_ vibronic features reflect the transitions from the first excited state to the ground state. (**b**) The intrachain exciton coherence number *α* of wire-bar-coated films is achieved by simulating PL spectra of films with Equation (2).

Figure 3a shows photoluminescence (PL) spectra of wire-bar-coated P3HT films, which are measured at 10 K. At 10 K, excitons are localized at the place where they are generated, allowing us to directly observe the phase morphology controlled exciton generation. Specifically, the PL spectrum of P3HT wire-bar-coated film shows the *I*_0-1_ dominated emission. However, the PL spectra of P3HT: PEO film represents the *I*_0-0_ dominated emission. This phenomenon means that the intrachain-induced aggregation was enhanced significantly in wire-bar-coated P3HT: PEO film, which is consistent with the increased *A*_0-0_ in their absorption spectra.

Quantitatively, based on these 10 K PL spectra, we further calculate the exciton coherence whether between or along the backbone of P3HT molecules of these films using the modified Franck–Condon model (given in Equation (2)),

(2) $$I\left(\omega \right)\propto {\left(\hbar \omega \right)}^{3}n{\left(\omega \right)}^{3}e^{-\lambda_{eff}{}^{2}}\left[\alpha \Gamma \left(\hbar \omega -E_{0}\right)+\underset{m=1,2\ldots }{\sum }\frac{\lambda_{eff}{}^{2m}}{m!}\Gamma \left(\hbar \omega -E_{0}+m\hbar \omega_{0}\right)\right]$$

Here, n(ω) is the real part of the refractive index of the film at the optical frequency ω, where $\lambda_{eff}{}^{2}$ is the effective Huang–Rhys factor (HR-factor), which describes the nuclear reorganization energy. The value of $\lambda_{eff}{}^{2}$ is fixed at 1, which is consistent with Equation (1). The parameter *α* quantifies the relative intensity of *I*_0-0_ vibronic progression. It is the exciton coherence number due to the competition between the intrachain and interchain exciton coupling, which has been uncoupled from the rest of the progression, indicating the intrachain coupling. *E*_0_ is the energy of the vibronic origin; $\hbar \omega_{0}$ is the vibronic spacing, which is the energy of the effective oscillator coupled to the electronic transition; m is the number of vibronic peaks; and Γ is a Gaussian function that represents the inhomogeneously broadened spectral line of the vibronic replica in the progression.

The simulated coherence number *α* of these films is plotted in Figure 3b. In the modified Franck–Condon model, the exciton coherence number *α* denotes the competition between the intrachain and interchain exciton coupling. If *α* is larger than 1, electronic spatial coherence is dominated by the intrachain coupling, implying a head-to-tail J-aggregate molecule packing. On the contrary, if *α* is smaller than 1, the spatial coherence of the film is dominated by the interchain coupling, indicating a side-by-side H-aggregate molecule packing. Thus, the exciton coherence number *α* can provide us with not only the type of exciton coupling but also the strength of the exciton coupling. The types and strength of exciton coupling have a profound influence on film optoelectronic processes.

It is evident that for P3HT wire-bar-coated film, the type of spatial coherence is the weakly interchain coherence, with *α* as 0.226, whereas in the case of P3HT: PEO wire-bar-coated film, the spatial coherence is dominated by the intrachain coupling, with *α* exceeding 1. Furthermore, as the bar sizes increased in the P3HT: PEO wire-bar-coated film, *α* increased sharply. Especially for 30 μm bar-coated film, *α* reached 1.4, indicating that intrachain coupling can be further enhanced in the J-aggregate regime. This implies that the exciton coherence length along the main backbone can be further increased, or the vibration coupling between the backbones can be further decreased in the J-aggregate regime by reducing the solvent solidification rate in film processing.

### 3.4. Exciton Dynamics in H- and J-Aggregate Regimes

**Figure 4:**
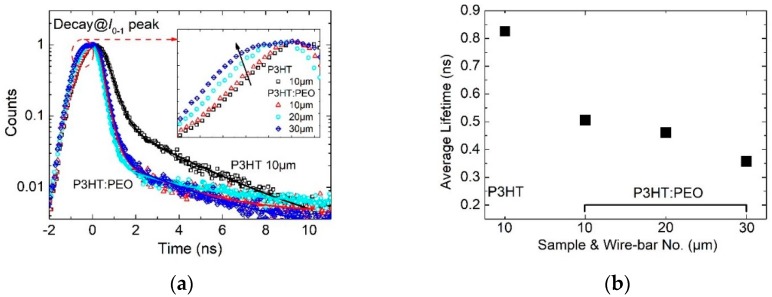
(**a**) Time-resolved PL spectra of poly (3-hexylthiophene-2,5-diyl) (P3HT) and P3HT: poly (ethylene oxide) (PEO) wire-bar-coated film with the excitation at 405 nm. Rising edges are magnified to provide the inset in (**a**). (**b**) Average lifetimes deduced from (**a**) are plotted as the function of bar sizes.

Next, we pay attention to exciton dynamics in H- and J-aggregate regimes to understand the influence of electronic coherence on their kinetics. Figure 4a shows the time-resolved PL spectra of P3HT and P3HT: PEO wire-bar-coated film. In the experiment, the film is excited at 405 nm and the dynamics are measured at their *I*_0-1_ emission peak. We find that the decay of P3HT film is the slowest one compared with that of P3HT: PEO film, whereas the decay of P3HT: PEO films are very similar.

Quantitatively, we used the second-order exponential function to fit these spectra (given in Equation (3)),

(3) $$I\left(t\right)=A+B_{1}e^{-\frac{t}{\tau_{1}}}+B_{2}e^{-\frac{t}{\tau_{2}}}$$

In order to neglect the excitation pulse duration, data are fitted after 60 ps, with the fitting results presented in Table 1. It has been demonstrated that the lifetime of short-lived excitons in the J-aggregate regime of P3HT molecules are ~ 50 ps [17], which is beyond our instrument responses (~60 ps). However, dramatically increased short lifetime species will influence the overall exciton lifetime. Thus, we calculated the average lifetime of excitons *τ*_average_ of these films by Equation (4),

(4) $$\tau_{\mathrm{average}}=\tau_{1}\frac{\tau_{1}B_{1}}{\tau_{1}B_{1}+\tau_{2}B_{2}}+\tau_{2}\frac{\tau_{2}B_{2}}{\tau_{1}B_{1}+\tau_{2}B_{2}}$$

The calculated average lifetime of these films are shown in Table 1 and plotted in Figure 4b, showing that the average lifetime is 0.826 ns for P3HT film, decreasing sharply to 0.506 ns for 10 μm P3HT: PEO films, and further decreasing for 20 μm (0.461 ns) and 30 μm (0.357 ns) P3HT: PEO films. These agree with the exciton coherence picture we put forward for the H- and J-aggregate regime. In the case of H-aggregate P3HT film, excitons cohere mainly across the polymer backbone, which has the larger free exciton bandwidth, and thus a longer lifetime. However, for the J-aggregate P3HT: PEO film, excitons cohere mainly along the polymer backbone, which holds the smaller free exciton bandwidth, and thus a shorter life.

However, if we look at the rising dynamics of these P3HT: PEO films, the counts or intensity increases slowly by increasing the bar numbers. Especially for the 30 μm bar-coated film, there is a noticeable intensity plateau on the photon accumulation dynamics (the rising dynamics before photons reach the maximum counts), as shown clearly in the inset in Figure 4a. We believe that there is a competition between the excitation (excitation pulse duration is ~60 ps) and the decay of short-lived exciton (~50 ps). This is another demonstration of the short lifetime exciton generation or recombination in the J-aggregate regime of P3HT molecules.

### 3.5. The Relationship between Processing Methods, Molecule Aggregation, Spatial Electronic Coherence, and Exciton Dynamics in H- and J-Aggregate Regimes

**Figure 5:**
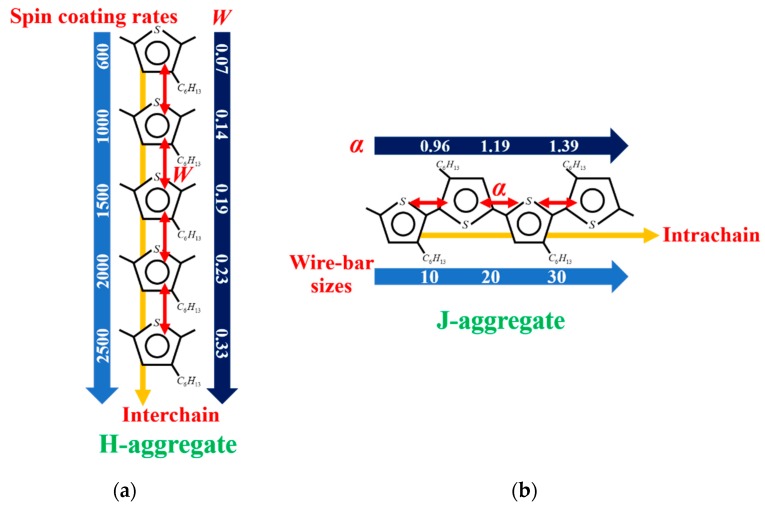
The schematic diagram of the relationship between processing methods, molecule aggregation, and the electronic spatial coherence of poly (3-hexylthiophene-2,5-diyl) (P3HT) film in H- (**a**) and J-aggregate (**b**) regimes, where *W* is free-exciton bandwidth and *α* is coherence number.

So far, we have achieved the different extent of short-range molecule aggregation in both H- and J-aggregate regimes in the P3HT film at room temperature. We also analyzed the different exciton coherence and dynamics based on these different molecule aggregation. Now, we are able to understand the interlink between the processing methods, molecule aggregation, and the electronic spatial coherence in H- and J-aggregate regimes, which are summarized as follows and shown in Figure 5.

In the H-aggregate regime:

(1) The formation of the H-aggregation of P3HT molecules requires a relatively fast solidification rate during film processing in order to prevent phase separation, such as spin-coated film in our investigation.
(2) Excitons in H-aggregated films denote a larger interchain free exciton bandwidth with *W* > 0 eV.
(3) The average lifetime of interchain-coherence-dominated excitons in H-aggregated films lasts longer, which is longer than 0.8 ns.

In the J-aggregate Regime:

(1) The formation of the J-aggregation of P3HT molecules requires a relatively slow solidification rate during the film processing in order to promote phase separation, such as wire-bar-coated film in our investigation.
(2) The excitons in J-aggregated film show a larger intrachain exciton coherence number with *α* > 1.
(3) The average lifetime of intrachain coherence dominated excitons in J-aggregated film is shorter (shorter than 0.5 ns).

## 4. Conclusions

We have successfully controlled the intrachain ordering/aggregation in the H- and J-aggregate regime in the semicrystalline conjugated polymer P3HT model systems. We have also demonstrated that the solidification rate during the film processing played an important role in the formation of molecule aggregation, which basically modified the phase separation of the film.

Based on the various spectroscopy investigations and the modified Franck–Condon model, we have further quantified the interchain coherence across the backbone (denoting as the free exciton bandwidth) and the intrachain coherence along the backbone (representing as the intrachain coherence number) in the H- and J-aggregate regimes.

We established the relationship between the processing method, the surface phase separation, the molecule aggregation, and the electronic spatial coherence of polymer P3HT. We believe that the intimate link between the processing method, the molecular aggregation, and spatial exciton coherence achieved from our model systems can generally apply to all other polymer systems, which play an important role in material design and polymer semiconductor device development.

## Figures and Tables

**Table 1:** The fitting results of P3HT film and P3HT: PEO film with different bar sizes.

Samples & Bar-Size	B_1_	*τ*_1_ (ns)	B_2_	*τ*_2_ (ns)	*τ*_average_ (ns)
P3HT (10 μm)	3.582	0.364	0.098	2.926	0.826
P3HT:PEO (10 μm)	2.375	0.261	0.027	2.640	0.506
P3HT:PEO (20 μm)	2.385	0.242	0.019	2.820	0.461
P3HT:PEO (30 μm)	4.690	0.254	0.046	1.820	0.357

## References

[1] Yao H., Li Y., Hu H., Chow P.C.Y., Chen S., Zhao J., Li Z., Carpenter J.H., Lai J.Y.L., Yang G. (2018). A Facile Method to Fine-Tune Polymer Aggregation Properties and Blend Morphology of Polymer Solar Cells Using Donor Polymers with Randomly Distributed Alkyl Chains. Adv. Energy Mater..

[2] Zhu L., Zhong W., Qiu C., Lyu B., Zhou Z., Zhang M., Song J., Xu J., Wang J., Ali J. (2019). Aggregation-Induced Multilength Scaled Morphology Enabling 11.76% Efficiency in All-Polymer Solar Cells Using Printing Fabrication. Adv. Mater..

[3] Liu Y., Zhao J., Li Z., Mu C., Ma W., Hu H., Jiang K., Lin H., Ade H., Yan H. (2014). Aggregation and morphology control enables multiple cases of high-efficiency polymer solar cells. Nat. Commun..

[4] Liu T., Pan X., Meng X., Liu Y., Wei D., Ma W., Huo L., Sun X., Lee T.H., Huang M. (2017). Alkyl Side-Chain Engineering in Wide-Bandgap Copolymers Leading to Power Conversion Efficiencies over 10%. Adv. Mater..

[5] Zhang Z.-G., Li Y. (2015). Side-chain engineering of high-efficiency conjugated polymer photovoltaic materials. Sci. China Chem..

[6] Lee C., Kang H., Lee W., Kim T., Kim K.-H., Woo H.Y., Wang C., Kim B.J. (2015). High-Performance All-Polymer Solar Cells Via Side-Chain Engineering of the Polymer Acceptor: The Importance of the Polymer Packing Structure and the Nanoscale Blend Morphology. Adv. Mater..

[7] Li S., Ye L., Zhao W., Yan H., Yang B., Liu D., Li W., Ade H., Hou J. (2018). A Wide Band Gap Polymer with a Deep Highest Occupied Molecular Orbital Level Enables 14.2% Efficiency in Polymer Solar Cells. J. Am. Chem. Soc..

[8] Hellmann C., Paquin F., Treat N.D., Bruno A., Reynolds L.X., Haque S.A., Stavrinou P.N., Silva C., Stingelin N. (2013). Controlling the interaction of light with polymer semiconductors. Adv. Mater..

[9] Hellmann C., Treat N.D., Scaccabarozzi A.D., Hollis J.R., Fleischli F.D., Bannock J.H., Mello J.D., Michels J.J., Kim J.-S., Stingelin N. (2015). Solution Processing of Polymer Semiconductors: Insulator Blends-Tailored Optical Properties through Liquid–Liquid Phase Separation Control. J. Polym. Sci. Polym. Phys..

[10] Pan S., Zhu M., He L., Zhang H., Qiu F., Lin Z., Peng J. (2018). Transformation from Nanofibers to Nanoribbons in Poly(3-hexylthiophene) Solution by Adding Alkylthiols. Macromol. Rapid Commun..

[11] Chang M., Lee J., Kleinhenz N., Fu B., Reichmanis E. (2014). Photoinduced Anisotropic Supramolecular Assembly and Enhanced Charge Transport of Poly(3-hexylthiophene) Thin Films. Adv. Funct. Mater..

[12] McDearmon B., Lim E., Lee I.-H., Kozycz L.M., O’Hara K., Robledo P.I., Venkatesan N.R., Chabinyc M.L., Hawker C.J. (2018). Effects of Side-Chain Topology on Aggregation of Conjugated Polymers. Macromolecules.

[13] Reichenberger M., Kroh D., Matrone G.M.M., Schötz K., Pröller S., Filonik O., Thordardottir M.E., Herzig E.M., Bässler H., Stingelin N. (2017). Controlling aggregate formation in conjugated polymers by spin-coating below the critical temperature of the disorder–order transition. J. Polym. Sci. Polym. Phys..

[14] Panzer F., Bässler H., Lohwasser R., Thelakkat M., Köhler A. (2014). The Impact of Polydispersity and Molecular Weight on the Order–Disorder Transition in Poly (3-hexylthiophene). J. Phys. Chem. Lett..

[15] Spano F.C., Silva C. (2014). H-and J-Aggregate Behavior in Polymeric Semiconductors. Annu. Rev. Phys. Chem..

[16] Clark J., Silva C., Friend R.H., Spano F.C. (2007). Role of Intermolecular Coupling in the Photophysics of Disordered Organic Semiconductors: Aggregate Emission in Regioregular Polythiophene. Phys. Rev. Lett..

[17] Wang H., Wang H.-Y., Gao B.-R., Wang L., Yang Z.-Y., Du X.-B., Chen Q.-D., Song J.-F., Sun H.-B. (2011). Exciton diffusion and charge transfer dynamics in nano phase-separated P3HT/PCBM blend films. Nanoscale.

